# The effect of minor allele frequency on the likelihood of obtaining false positives

**DOI:** 10.1186/1753-6561-3-S7-S41

**Published:** 2009-12-15

**Authors:** Meredith E Tabangin, Jessica G Woo, Lisa J Martin

**Affiliations:** 1 Division of Biostatistics and Epidemiology, Cincinnati Children's Hospital Medical Center, 3333 Burnet Avenue, Mail Code 5041, Cincinnati, Ohio 45229, USA; 2 The University of Cincinnati College of Medicine, 231 Albert Sabin Way, Cincinnati, Ohio 45267, USA

## Abstract

Determining the most promising single-nucleotide polymorphisms (SNPs) presents a challenge in genome-wide association studies, when hundreds of thousands of association tests are conducted. The power to detect genetic effects is dependent on minor allele frequency (MAF), and genome-wide association studies SNP arrays include SNPs with a wide distribution of MAFs. Therefore, it is critical to understand MAF's effect on the false positive rate.

Data from the Framingham Heart Study simulated data (Problem 3, with answers) was used to examine the effects of varying MAFs on the likelihood of false positives. Replication set 1 was used to generate 1 million permutations of case/control status in unrelated individuals. Logistic regression was used to test for the association between each SNP and myocardial infarction using an additive model. We report the number of "significant" tests by MAF at α = 10^-4^, 10^-5^, and 10^-6^.

Common SNPs exhibited fewer false positives than expected. At α = 10^-4^, SNPs with MAF 25% and 50% resulted in 69.2 [95%CI: 62.8-75.6] and 70.8 [95%CI: 61.3-80.4] false positives, respectively, compared to 100 expected. Rare SNPs exhibited more variability but did not show more false-positive results than expected by chance. However, at α = 10^-4^, MAF = 5% exhibited significantly more false positives (105.5 [95%CI: 81-130.1]) than MAF = 25% and 50%. Similar results were seen at the other alpha values.

These results suggest that removal of low MAF SNPs from analysis due to concerns about inflated false-positive results may not be appropriate.

## Background

Correct identification of the most promising single-nucleotide polymorphisms (SNPs) for follow-up is one of the greatest challenges of conducting genome-wide association studies (GWAS). Hundreds of thousands of simultaneous association tests are typically conducted across the genome, and the resulting *p*-values or test statistics are compared and often ranked to determine the most promising SNPs. This procedure is conducted regardless of the specific adjustment chosen for multiple testing correction. For example, the conservative Bonferroni correction to adjust the critical significance threshold simply lowers the threshold of α to account for the number of tests conducted; any SNP with a *p*-value below that adjusted threshold will be considered significant. False-discovery rate (FDR) procedures explicitly rank *p*-values before selecting the SNPs below the FDR threshold for further consideration. Genome-wide permutation testing methods involve randomly permuting case and control status and examining the distribution of the best resulting test statistic, thus creating an empirical distribution of extreme test statistics against which to compare observed results. With each of these methods however, if multiple SNPs reach this threshold, the investigator must choose which SNPs to follow for replication.

GWAS SNP arrays necessarily include SNPs with a wide distribution of minor allele frequencies (MAFs), from nearly monomorphic (MAF <0.5%) to very common (MAF≈50%). All of the methods for determining SNP selection implicitly assume that the *p*-values or test statistics from individual SNPs are drawn from the same distribution, regardless of MAF. If this were true, then ranking the test statistics from SNPs of different MAF would be valid. However, the power to detect a given genetic effect with a given study size depends to a great extent on the MAF of the risk allele tested. Specifically, loci with a low MAF (<10%) have significantly lower power to detect weak genotypic risk ratios than loci with a high MAF (>40%) [1]. Further, previous studies have demonstrated that rare genotypes are more likely to result in spurious findings [2]. Thus, many GWAS have removed SNPs with MAF<10% [3,4].

It is therefore critical to understand the true effect of MAF on the likelihood of obtaining a false-positive result (e.g., very low *p*-value) under the null hypothesis of no true association. We hypothesize that MAF affects the likelihood of a specific variant achieving significance thresholds under the null hypothesis. If MAF does affect the probability of achieving a specific significance, this would result in non-comparability of *p*-values (or test statistics) between SNPs with different MAFs. To address this issue we utilized the Genetic Analysis Workshop 16 simulated data set and performed extensive permutations.

## Methods

The Framingham Heart Study simulated data set (Problem 3, replication 1, with answers) was used in this analysis. Founders and singletons from the original pedigree file (*N *= 849) were selected for this case-control analysis, using myocardial infarction (event 1) as the disease status, resulting in 133 cases and 716 controls. SNPs were eligible for selection if they were located in genomic regions not associated with myocardial infarction (null SNPs), of which 50 were selected by MAF and percent missing data. Ten SNPs, each at lower MAFs of 1% and 5%, and five SNPs, each at MAFs of 10%, 25%, and 50%, with similar low rates of missing data (approximately 0%) were selected. To examine effects of missing data, ten additional SNPs at MAF 5% and five additional SNPs each at MAF 50% with approximately 5% missing data were also analyzed (Table 1).

**Table 1:** SNP Selection

MAF 1%		MAF 5%		MAF 10%		MAF 25%		MAF 50%	
**SNP**	**% Missing**	**SNP**	**% Missing**	**SNP**	**% Missing**	**SNP**	**% Missing**	**SNP**	**% Missing**

rs1722601	0	rs6000509	0.06	rs7291198	0.04	rs2413030	0.01	rs165862	0
rs16991890	0.10	rs7289964	0.45	rs2227235	0.10	rs1139793	0.16	rs695670	0.01
rs6001118	0.28	rs9622750	0.16	rs17299838	0.28	rs2071436	0.12	rs5998941	0
rs12157495	0.09	rs6518774	0.12	rs730483	0.04	rs5752905	0.06	rs5758972	0.01
rs16994023	0	rs8139704	0.18	rs9615812	0.03	rs5753917	0.07	rs738536	0
rs5993835	0	rs134049	0					rs2267296	5.67
rs16997431	0.01	rs13058493	0					rs136575	5.29
rs17004911	0	rs16989600	0					rs2254907	4.73
rs7289701	0	rs5998473	0					rs2179321	5.45
rs11912108	0	rs2267613	0					rs6120328	5.65
		rs1110477	4.50						
		rs1476053	4.60						
		rs16998312	4.75						
		rs17808497	4.76						
		rs17762006	5.29						
		rs6082126	5.27						
		rs11698699	5.33						
		rs8120594	4.98						
		rs16989639	5.33						
		rs6123569	5.30						

One million permutations were conducted per SNP, in which case or control status was randomly assigned to an individual in the dataset, keeping the total numbers of cases and controls consistent. For each permutation, logistic regression was used to test for the association between each SNP and myocardial infarction event 1 using an additive model. To determine whether the test statistic distribution differs by allele frequency we report the number of false positive "significant" tests by MAF at α = 10^-4^, 10^-5^, or 10^-6^. These threshold alpha values were selected because we could expect at least one false positive by chance using one million permutations. Statistical analysis was conducted using SAS version 9.1.3 (SAS Institute, Cary, NC) on a dedicated server.

## Results

**Figure 1:**
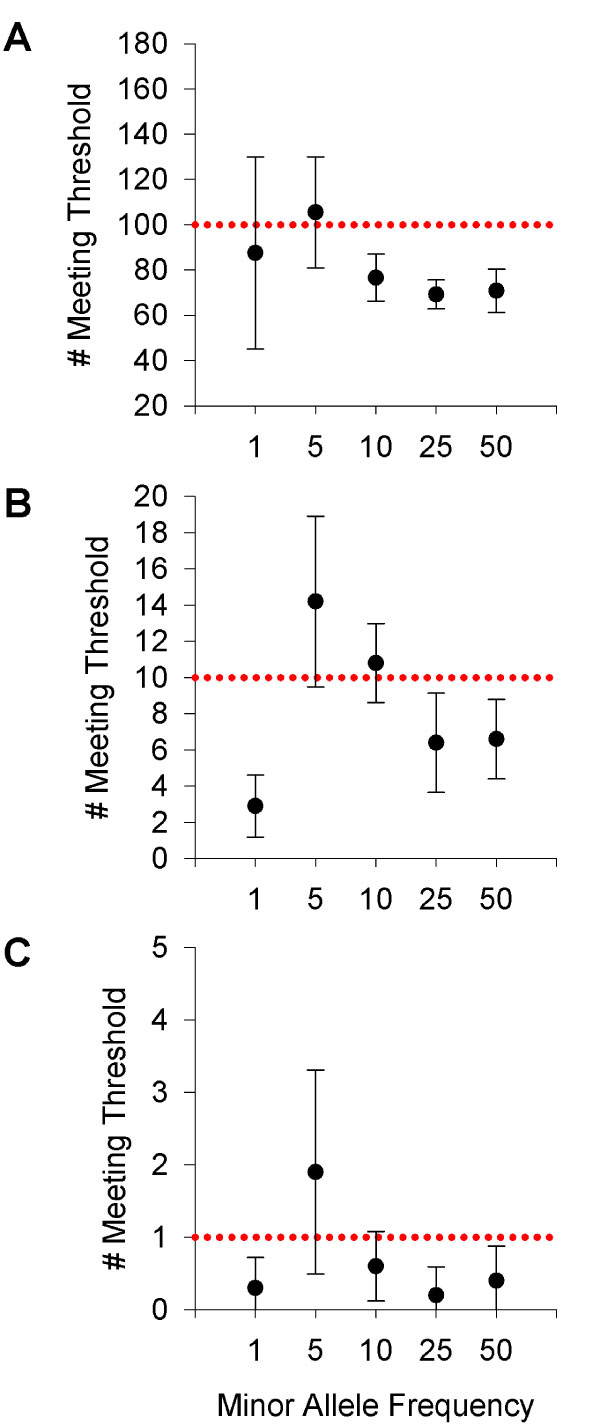
**Permuted runs reaching significance**. Means ± 95% confidence intervals of number of permuted runs reaching significance at α ≤ 10^-4 ^(Panel A), α ≤ 10^-5 ^(Panel B), and α ≤ 10^-6 ^(Panel C). The dotted line in each panel represents the expected number of significant tests expected by chance.

Common SNPs (MAF>25%) exhibited significantly fewer false positives than expected by chance at all thresholds tested (e.g., 10^-4^, 10^-5^, and 10^-6^; Figure 1). Rare SNPs exhibited more variability in the number of false positives than common SNPs, but did not have significantly more of false-positive results than expected by chance. Interestingly, when SNPs with 5% missing data were analyzed, the findings did not differ from the results using SNPs with almost no missing data (data not shown). Given the marked variability in the 1% MAF, the relationship between numbers of individuals homozygous for the 1% allele and number of false positives was examined, but no trend was present.

While low MAF SNPs (1% or 5%) did not have elevated false positives compared to chance, the 5% MAF SNPs had significantly more false positives than the more common (25 and 50%) MAF SNPs at the 10^-4 ^(*p *< 0.03) and 10^-5 ^(*p *< 0.05)thresholds. These differences were not captured by standard summary statistics, but instead, only by examining values at the tail end of the distribution.

## Discussion

The current study provides evidence that false-positive rate is influenced by MAF. We report that common SNPs (MAF 25-50%) result in significantly fewer false positives than expected under the null χ^2 ^distribution, suggesting that for common SNPs the current thresholds may be too conservative. Also, we report more false positives with 5% MAF SNPs than with common SNPs at the thresholds examined. In addition, under the null hypothesis, up to 5% randomly missing data did not impact our findings. Thus, under the null hypothesis, MAF but not proportion of missing data appears to affect the likelihood of a false positive result.

These results have implications for a common analytical approach applied to large-scale SNP data. GWAS analysis often begins by discarding all genotypes for SNPs of low MAF, typically for those with MAF<10% [3,4]. This practice results in a huge loss of data. For example, 35.5% of SNPs on the Affymetrix chips have MAFs below 10%. In addition, discarding low MAF SNPs may hamper our ability to detect rare disease-causing polymorphisms [5].

The reasons for discarding these SNPs may be related to genotyping issues, such as lower genotyping rates or concerns about calling accuracy. However, discarding low-MAF SNPs may also be due to perceptions about the statistical inferences that result from analyzing such SNPs. Anecdotally, investigators have expressed "distrust" of significant results arising from low-MAF SNPs. However, a recent study demonstrated that in the absence of bias in genotype error rates between cases and controls, even very small MAFs show type I error rates close to nominal levels [6]. Our study confirms and extends this report, showing that nominally significant results occur significantly less often than expected, even for low MAF SNPs, resulting in a conservative bias. Thus, our findings do not support the removal of low MAF SNPs from analysis due to concerns about inflated false-positive results.

However, our study does raise the concern about ranking *p*-values generated from SNPs across vastly different MAF. Given our findings, we suggest that the χ^2 ^threshold to establish a particular level of significance may need to be modified using empirical permutations for several MAF "bins." For example, in our analysis dataset, for an MAF of 25% and a significance threshold of 10^-5^, the threshold χ^2 ^value would need to be reduced from 19.51 (from the standard χ^2 ^distribution) to 18.79 to capture the expected 10 false positives. However, it should be noted that to determine robust estimates, χ^2 ^adjustments, and false positive rates for a GWAS significance of 10^-8^, over 10 billion permutations and significant computing capability would be required. Additional studies using larger datasets will be required to fully understand this issue.

## List of abbreviations used

FDR: False discovery rate; GWAS: Genome-wide association studies; MAF: Minor allele frequency; SNP: Single-nucleotide polymorphism.

## Competing interests

The authors declare that they have no competing interests.

## Authors' contributions

MT participated in the design of the study, performed the statistical analysis, and helped to draft the manuscript. JW and LM conceived of the study, and participated in its design and coordination and helped to draft the manuscript. All authors read and approved the final manuscript.
